# Outbreak of *Wickerhamomyces anomalus* (formerly *Candida pelliculosa*) Bloodstream Infections, Venezuela, 2022–2023^1^

**DOI:** 10.3201/eid3206.251978

**Published:** 2026-06

**Authors:** Maribel Dolande-Franco, Diego H. Caceres, Juan F. Frey-Carrillo, Antoniellys Pérez-Guzmán, Andres Ceballos-Garzon, Aleiram Chaurio-Briceño, Bram Spruijtenburg, Yusely Roa-Díaz, Eelco F.J. Meijer, Elaine Cristina Francisco, Rodrigo Oliveira, Tatiana Drummond-Suinaga, Jacques F. Meis

**Affiliations:** Instituto Nacional de Higiene “Rafael Rangel,” Caracas, Venezuela (M. Dolande-Franco, J.F. Frey-Carrillo, A. Chaurio-Briceño); Immuno-Mycologics, Norman, Oklahoma, USA (D.H. Caceres); Universidad del Rosario, Bogota, Colombia (D.H. Caceres, A. Ceballos-Garzon); Radboudumc-CWZ Center of Expertise for Mycology, Nijmegen, the Netherlands (D.H. Caceres, B. Spruijtenburg, E.F.J. Meijer, J.F. Meis); Hospital Universitario de Caracas, Caracas (A. Pérez-Guzmán, Y. Roa-Díaz, T. Drummond-Suinaga); Canisius-Wilhelmina Hospital/Dicoon, Nijmegen (B. Spruijtenburg, E.F.J. Meijer); Universidade Federal de São Paulo, São Paulo, Brazil (E.C. Francisco); Antimicrobial Resistance Institute of São Paulo, São Paulo (E.C. Francisco); Universidade Federal do Paraná, Curitiba, Brazil (E.C. Francisco, J.F. Meis); Bruker Daltonics GmbH & Co., Bremen, Germany (R. Oliveira); University of Cologne, Cologne, Germany (J.F. Meis)

**Keywords:** fungi, outbreak, *Wickerhamomyces anomalus*, bloodstream infections, candidemia, Venezuela, rare yeast pathogens, invasive fungal infections

## Abstract

During August 2022–December 2023, a total of 110 bloodstream infections caused by *Wickerhamomyces anomalus* (synonym *Candida pelliculosa*) were identified across 8 hospitals in 3 cities in Venezuela. Most cases (82/110 in Caracas) occurred in a single pediatric intensive care unit, predominantly among neonates. Molecular genotyping indicated multiple events of clonal transmission, which was supported by epidemiologic clustering of patients. Antifungal susceptibility testing demonstrated good in vitro activity; most isolates were classified as wild-type. Our findings underscore the need for enhanced fungal diagnostics, infection prevention measures, and national surveillance to mitigate hospital-associated fungal transmission in resource-limited settings.

*Wickerhamomyces anomalus* (synonyms *Hansenula anomala*, *Candida pelliculosa*, and *Pichia anomala*) has been isolated from soil, grains, fruit juices, and animals (*1*). In agriculture, *W. anomalus* has been used in beer production and for fermentation of cocoa and coffee beans and is responsible for spoilage of bakery products (*2*). *W. anomalus* is also used in the biocontrol of molds, especially those that affect stored postharvest products (*2*). Although use in food and industrial applications is common, clinically, *W. anomalus* is considered a rare emerging pathogen (*3*). Invasive disease by this agent has been reported mainly in low-birthweight infants admitted to neonatal intensive care units (NICUs) (*3*–*9*), occasionally in adult immunocompromised patients (*10*,*11*), and as a cause of keratitis (*12*). The mortality rate for *W. anomalus* fungemia is estimated at 30%, comparable to other yeast pathogens in neonates (*6*). Although antifungal resistance is on the rise for several notorious yeasts like *C. auris* and *C. parapsilosis*, it appears to be rare for *W. anomalus* (*8*,*13*).

Of note, nosocomial clonal outbreaks have been reported mainly in NICUs or pediatric intensive care units (PICUs) (*1*,*14*). When *W. anomalus* fungemia is diagnosed in multiple patients in the same healthcare center, rapid and high-resolution genotyping is required to identify the source and prevent future cases (*15*). In this article, we report a large *W. anomalus* fungemia outbreak spread across several healthcare facilities in Venezuela and apply descriptive epidemiology, short tandem repeat (STR) analysis, and antifungal susceptibility testing (AFST) to characterize the outbreak.

## Material and Methods

**Figure 1:**
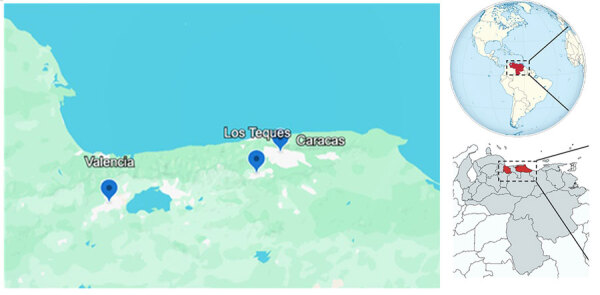
Geolocations of reported *Wickerhamomyces anomalus* (formerly *Candida pelliculosa*) bloodstream infection outbreaks, Venezuela, 2022–2023. The map shows the distribution of outbreaks across 8 hospitals in 3 cities: Caracas (Capital District; n = 5 hospitals), Los Teques (Miranda State; n = 1 hospital), and Valencia (Carabobo State; n = 2 hospitals). Caracas, located near Venezuela’s northern coast, represents the primary cluster. Los Teques lies ≈29 km southwest of Caracas, and Valencia is located ≈177 km west of Caracas. The geographic dispersion and clustering across multiple healthcare facilities are consistent with regional nosocomial transmission rather than a single point-source event. Insets show location of the 3 states in Venezuela and the location of Venezuela in South America.

We conducted a descriptive, retrospective investigation of bloodstream infections (BSIs) caused by *W. anomalus* in Venezuela during August 2022–December 2023. We defined a confirmed case as a patient with *W. anomalus* isolated from a blood culture in the presence of compatible clinical signs and symptoms of infection. We defined clinical criteria for suspected fungemia on the basis of established risk factors, such as prematurity, low birthweight, prolonged exposure to broad-spectrum antimicrobial agents, presence of a central venous catheter, and receipt of mechanical ventilation. Case notifications were received through the National Mycology Reference Network from 8 hospitals across 3 cities: Caracas (Capital District), Los Teques (Miranda state), and Valencia (Carabobo state). Caracas, in the Capital District, lies near Venezuela’s northern coast; Los Teques (Miranda) is ≈18 miles (29 km) southwest of Caracas; and Valencia (Carabobo) is ≈110 miles (177 km) west of Caracas (Figure 1).

We extracted available demographic, clinical, and epidemiologic data from laboratory and hospital records where accessible. Clinical information was not uniformly available and could only be retrieved for a subset of cases. Case counts were plotted by epidemiologic week (EW) and location to describe temporal and spatial clustering. Because of the retrospective nature of the study, real-time intervention throughout the course of the outbreak was not feasible. We did not perform environmental sampling; the investigation was part of a national public health response and was therefore exempt from institutional review board approval.

### Isolates 

From a total of 110 documented cases of *W. anomalus* BSIs, 99 isolates from unique patients were available for this study. All isolates, initially identified using the automated VITEK 2 system (bioMérieux, https://www.biomerieux.com) (Appendix Table 1), were stored according to standard procedures at −80°C. Isolates were grown on Sabouraud dextrose agar plates (Thermo Fisher Scientific, https://www.thermofisher.com) at 30°C for 1 day before confirmation of identification using matrix-assisted laser desorption ionization-time of flight (MALDI-TOF) mass spectrometry as previously described (*14*).

### MALDI-TOF Mass Spectrometry Identification and Data Analysis 

We identified isolates using MALDI-TOF mass spectrometry. We performed protein extraction following the standard procedures recommended by Bruker (https://www.bruker.com). We acquired protein spectra using the MALDI Biotyper Sirius System, and identifications were generated with the MBT Compass HT IVD database (Bruker).

In addition to species-level identification, we further analyzed protein spectral data in MBT Compass Explorer to evaluate spectral relatedness among isolates. This analysis included the generation of similarity matrices and dendrograms using the software’s integrated data-processing algorithms.

### STR Genotyping 

We extracted DNA from all cultured isolates with the MagNA Pure 96 instrument and MagNA Pure DNA and Viral NA Small volume kit (Roche, https://www.roche.com) according to the manufacturer’s instruction, as described previously (*16*). We performed multiplex PCR reaction amplifying 6 previously described STR markers using a thermocycler (Analytik Jena, https://www.analytik-jena.us) under identical PCR conditions (*14*). We analyzed amplicons on a 3500 XL genetic analyzer (Thermo Fisher Scientific), determined copy numbers using GeneMapper version 5 software (Thermo Fisher Scientific), and inferred phylogenetic relatedness between isolates using BioNumerics version 7.6.1 (bioMérieux, https://www.biomerieux.com) (*14*).

### Antifungal Susceptibility Testing 

We initially performed AFST using VITEK 2 AST-YS01 cards (bioMérieux) and confirmed results using microbroth dilution Clinical and Laboratory Standards Institute (CLSI) reference standard M27 (*17*) using amphotericin B (Bristol Myers Squib, https://www.bms.com), fluconazole (Merck, https://www.merck.com), itraconazole (Johnson & Johnson, https://www.jnj.com), voriconazole (Pfizer, https://www.pfizer.com), posaconazole (Merck), isavuconazole (Merck), anidulafungin (Merck), and micafungin (Merck). We incubated microtiter plates at 35°C and visually interpreted them after 24 hours. We read MICs as the lowest antifungal concentration with a 50% growth reduction when compared with the growth control, except for amphotericin B with 100% growth reduction. We interpreted MICs according to epidemiologic cutoff values (ECVs) established in CLSI reference standard M57S (*18*), specifically 1 µg/mL for amphotericin B, 8 µg/mL for fluconazole, 1 µg/mL for itraconazole, 0.12 µg/mL for micafungin, 2 µg/mL for posaconazole, and 0.25 µg/mL for voriconazole.

## Results

### Chronological Narrative of Outbreak 

**Figure 2:**
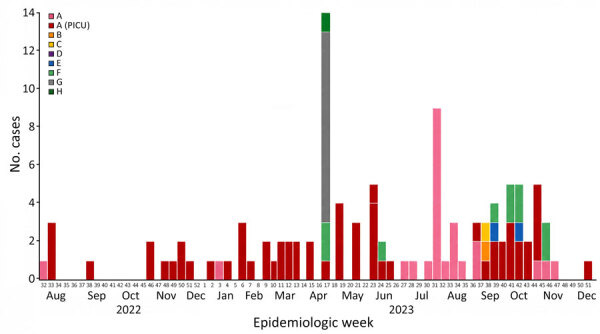
Epidemic curve of outbreak of *Wickerhamomyces anomalus* (formerly *Candida pelliculosa*) bloodstream infections, Venezuela, 2022–2023. Curve is shown for 110 cases by epidemiologic week, with cases color-coded according to the medical center of origin (hospitals A–H). The distribution highlights temporal clustering of cases across multiple institutions, including a sustained cluster in the hospital A PICU and shorter, city-specific clusters in other centers. PICU, pediatric intensive care unit.

In August 2022, the National Mycology Reference Network in Venezuela received notifications of an unusual increase in yeast bloodstream isolates from the PICU at a public hospital in Caracas (hospital A). This signal was identified through routine laboratory-based surveillance, in a context where clinical and epidemiologic data were not systematically captured or shared with the national network. The first case of BSI caused by *W. anomalus* was identified during EW 32 of 2022. During August 2022–December 2023, a total of 110 confirmed BSI cases were identified from 3 cities: Caracas (n = 88 cases), Los Teques (n = 11 cases), and Valencia (n = 11 cases) (Figure 2). Given the retrospective nature of this investigation, outbreak recognition and characterization occurred after transmission had already been established, and no coordinated real-time public health or infection prevention and control interventions were implemented consistently throughout the course of the outbreak.

In Caracas, 5 hospitals were affected (hospitals A–E); most cases (82 of 88) were concentrated at hospital A. Among Caracas cases, 61 (74%) cases occurred in the NICU/PICU, primarily among neonates (<30 days of age). Although detailed clinical data were only available for a subset of patients, the concentration of cases in high-risk units and the temporal clustering strongly support a nosocomial transmission pattern, consistent with previous reports of *W. anomalus* outbreaks in NICU and PICU settings.

At hospital A, PICU cases occurred in an initial cluster from EW 32 of 2022 through EW 25 of 2023 (43 cases), and 1 case occurred outside the PICU during 2023 EW 3. A second cluster occurred outside the PICU from EW 27 through EW 36 of 2023 (17 cases), followed by 2 additional clusters: 1 in the PICU during EWs 36–43 of 2023 (17 cases), another outside the PICU during EWs 43–45 of 2023 (3 cases), and 1 case at the PICU during EW 51 (Figure 2). The remaining 6 cases from Caracas were distributed among other hospitals: 1 case at hospital B during EW 23 of 2023, 1 case at hospital C during EW 31 of 2023, 1 case at hospital D during EW 32 of 2023, and a cluster of 3 cases at hospital E during EWs 37–41 of 2023 (Figure 2).

In Los Teques, 11 cases were reported from hospital F. The first 2 cases were identified during EW 17 of 2023, followed by 1 sporadic case in EW 24 of 2023 and a subsequent cluster of 8 cases during EWs 37–44 of 2023 (Figure 2). In Valencia, 11 cases were reported during EW 11 of 2023. Ten cases were reported from hospital G, and 1 case was reported from hospital H (Figure 2).

### Epidemiologic Investigation 

The index case was reported in week 32 of 2022 (Figure 2). Beginning in week 46 of 2022, cases were reported at least every 3 weeks; 2 peaks occurred in weeks 17 and 31 of 2023. The first peak occurred predominantly in hospital G in Valencia; those were the only reported cases in that particular week. Hospital A in Caracas reported cases throughout the entire study period. Although cases were initially restricted to the PICU, beginning in week 27 of 2023, the yeast was also found in non–intensive care wards. The investigation suggested nosocomial transmission within NICUs and PICUs. The clustering of cases by hospital unit and time strongly suggests horizontal transmission, most likely occurring through contaminated hands, infusion fluids, or reusable medical equipment. Despite the absence of comprehensive clinical and environmental data, the observed temporal clustering, concentration in specific hospital units, and spread across institutions are consistent with an actual outbreak rather than a pseudo-outbreak, most likely driven by healthcare-associated transmission mechanisms.

### Patient Demographics and Clinical Characteristics 

Among the 110 patients, 68 (62%) were male and 42 (38%) female. The median age was 11 days (interquartile range 8–22 days). Most patients (99 [90%]) were neonates (0–30 days of age), 6 (5%) were infants (31–365 days of age), 4 (4%) were preschool-aged children (1–5 years), and 1 (1%) was an adult (a 37-year-old man).

Since January 20, 2023, *Candida* and other yeast infections have been included in the list of notifiable epidemiologic events in Venezuela (https://mpps.gob.ve/wp-content/uploads/2023/02/G.O.-42.553-20012023-ENO.pdf). However, at the time this outbreak occurred, surveillance was exclusively laboratory-based, and no standardized instrument existed for reporting epidemiologic or clinical data. Of the 110 cases identified, epidemiologic and clinical information was available for only 15 patients, all of whom were admitted to the PICU of hospital A. Those cases occurred from EW 32 of 2022 through EW 17 of 2023. In 8 of the 15 cases, we did not confirm identification by MALDI-TOF mass spectrometry or sequencing. The predominant risk factors among those 15 patients were PICU admission, prematurity, and the presence of central venous catheters, all of which were reported in all cases. Hematologic abnormalities were frequent; thrombocytopenia was observed in all patients, followed by leukocytosis (10/15), anemia (6/15), and leukopenia (2/15).

Of the 15 patients, 7 initially received fluconazole at 12 mg/kg/day. In 6 of those cases, a change in antifungal treatment was required: 1 patient was switched to caspofungin (loading dose 70 mg/m^2^/d, maintenance dose 50 mg/m^2^/d, administered over 1 h for 14 d) and 5 were switched to deoxycholate amphotericin B (1 mg/kg/d, infused over 3 h). Among the 5 patients treated with deoxycholate amphotericin B, 1 died; however, the antifungal response in this case was favorable, and death was attributed to complications of the underlying disease rather than to fungal infection.

In the remaining 8 patients, deoxycholate amphotericin B was used as the initial antifungal therapy. In 1 of those patients, treatment was subsequently changed from deoxycholate amphotericin B to caspofungin. Of the 8 patients who started therapy with deoxycholate amphotericin B, 1 died because of underlying medical conditions; another patient initially treated with deoxycholate amphotericin B and later switched to caspofungin died from causes directly associated with persistent candidemia.

### Microbiological Findings 

We performed initial identification of the isolates using the automated VITEK 2 system (bioMérieux), which identified all isolates as *C. pelliculosa* (*W. anomalus*). Because this identification was uncommon, we sent viable isolates (n = 99) to the Department of Medical Microbiology and Immunology, Canisius-Wilhelmina Hospital (CWZ)/Dicoon, Nijmegen, the Netherlands, for confirmation by MALDI-TOF mass spectrometry and molecular analysis.

Overall, the results obtained with the VITEK 2 system (bioMérieux) were consistent with those from MALDI-TOF MS; all isolates were accurately identified as *W. anomalus* (log-score >2.0). Using the VITEK 2 AST-YS01 cards, 4 (4%) of 99 isolates exhibited a MIC >32 µg/mL for fluconazole, 18 (18%) exhibited a MIC >0.25 µg/mL for voriconazole, and 4 (4%) had a MIC >2 µg/mL for amphotericin B. We subsequently tested the 99 *W. anomalus* isolates with CLSI broth microdilution against 8 antifungals, demonstrating major discrepancies with the VITEK 2 system (bioMérieux). All antifungals demonstrated potent in vitro activity; echinocandins showed the lowest MIC_50_ of <0.008 µg/mL (Table). For the azoles, isavuconazole had the highest in vitro activity with a MIC_50_ of 0.031 µg/mL. Because breakpoints are absent for *W. anomalus*, available CLSI ECVs were applied, resulting in classification of 1 isolate (L-13) as non–wild-type for fluconazole (MIC 8 µg/mL) and 1 isolate (L-03) as non–wild-type for voriconazole (MIC 0.25 µg/mL), although the MICs of both isolates were at the cutoff value. For amphotericin B, itraconazole, micafungin, and posaconazole, all isolates were classified as wild-type (Appendix Table 2).

**Table:** In vitro antifungal susceptibility testing metrics of 99 *Wickerhamomyces anomalus* isolates in study of outbreak of *W. anomalus* (formerly *Candida pelliculosa*) bloodstream infections, Venezuela, 2022–2023*

Antifungal	Range	Geometric mean	MIC_50_	MIC_90_
Amphotericin B	0.063–0.25	0.077	0.063	0.125
Fluconazole	1–8	1.99	2	2
Itraconazole	0.031–0.25	0.068	0.063	0.125
Voriconazole	0.031–0.25	0.064	0.063	0.125
Posaconazole	0.031–0.25	0.059	0.063	0.063
Isavuconazole	0.016–0.125	0.029	0.031	0.063
Anidulafungin	≤0.008–0.031	0.01	≤0.008	0.016
Micafungin	≤0.008–0.063	0.012	≤0.008	0.031

*Values are µg/mL. In vitro antifungal susceptibility testing was conducted using the Clinical and Laboratory Standards Institute M27 broth microdilution (*17*). MIC_50_, MIC that inhibits 50% of isolates; MIC_90,_ MIC that inhibits 90% of isolates.

### Outbreak Investigation 

**Figure 3:**
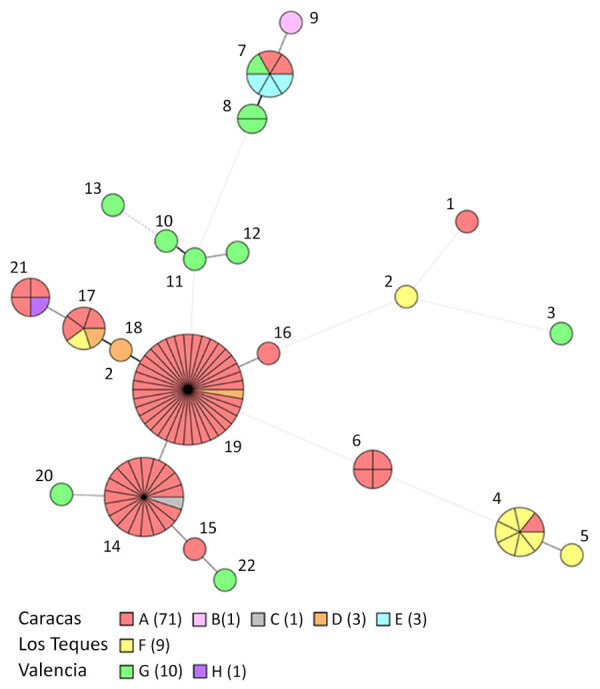
Short tandem repeat genotyping of 99 *Wickerhamomyces anomalus* isolates in study of outbreak of *W. anomalus* (formerly *Candida pelliculosa*) bloodstream infections, Venezuela, 2022–2023. Minimum-spanning tree of 99 *W. anomalus* isolates based on microsatellite markers. Branch lengths indicate similarity between isolates with thick solid lines (variation in 1 allele), thin solid lines (variation in 2 alleles), and thin dotted lines (variation in >4 alleles). Isolates are colored according to the city and hospital of origin; parenthetical values in the legend represent the number of isolates.

We assessed genetic relatedness between isolates with STR genotyping for all 99 *W. anomalus* isolates, resulting in 22 genotypes with 7 large clusters that consisted of 4–39 isolates (Figure 3). Only 1 large cluster (genotype 6) was confined to a single center; smaller clusters were found in 2–3 centers. Only genotypes 14 and 19 were found in both 2022 and 2023, whereas the other clusters were found exclusively in 2023.

**Figure 4:**
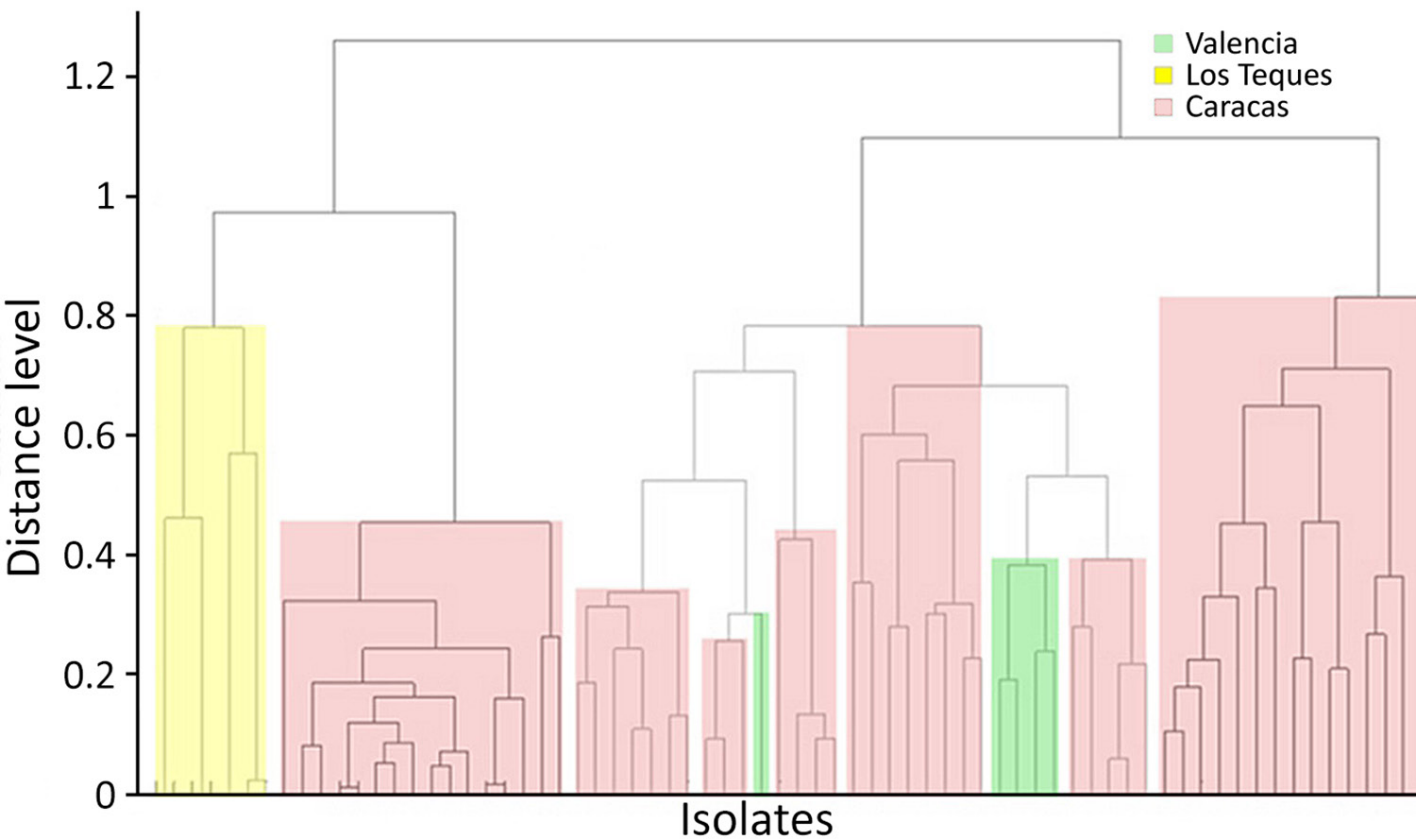
Matrix-assisted laser desorption/ionization time-of-flight mass spectrometry–based dendrogram showing spectral relatedness among *Wickerhamomyces anomalus* isolates in study of outbreak of *W. anomalus* (formerly *Candida pelliculosa*) bloodstream infections, Venezuela, 2022–2023. Clustering reflects geographic origin.

MALDI-TOF mass spectrometry–based dendrogram analysis also separated *W. anomalus* isolates according to geographic origin. Los Teques isolates formed a tight and highly similar cluster, whereas Valencia isolates showed higher heterogeneity, including 1 isolate forming a distinct group and the remaining isolates clustering within a small branch that further resolved into different subclusters. In contrast, isolates from Caracas displayed broader dispersion across multiple subclusters, highlighting greater heterogeneity within this region. Concordance between molecular (STR) and proteomic (MALDI-TOF) approaches supports the robustness of the observed population structure and highlights the predominance of a major Caracas-associated clone (Figure 4).

## Discussion

This investigation describes an outbreak of *W. anomalus* BSIs involving 110 patients across 3 cities and 8 hospitals in Venezuela during 2022–2023. A pseudo-outbreak was ruled out on the basis of several findings. Cases occurred over an extended period with recurrent unit-level clustering, involved multiple hospitals and cities, and affected patients with consistent clinical and laboratory features suggestive of true infection. In addition, the identification of multiple genotypes supports nosocomial transmission rather than a single contamination source, making a pseudo-outbreak unlikely. Previous reports of *W. anomalus* outbreaks describe relatively small clusters, typically ranging from 5 to 20 cases of BSIs, except for a large outbreak from India consisting of 379 cases (*4*). Most reported cases occurred in NICU or pediatric wards in Asia (India, China, Korea, Taiwan, and Pakistan) and Latin America (Brazil) (*1*,*4*–*9*,*11*,*19*–*21*). Despite its reported low virulence, *W. anomalus* can cause severe infections in highly vulnerable patients, particularly preterm neonates and critically ill children exposed to invasive medical procedures (*1*,*4*–*9*,*11*,*19*–*21*).

The marked predominance of neonatal cases and the clustering within PICUs and NICUs in the outbreak we report strongly support a pattern of hospital-associated transmission. Similar outbreaks have previously been linked to contaminated intravenous fluids, parenteral nutrition, reusable medical equipment, or healthcare workers’ hands (*1*,*4*,*6*–*9*,*19*,*21*). In this outbreak, the temporal and spatial aggregation of cases with an uncommon yeast within specific hospital units, along with molecular evidence of clonal relatedness as shown by STR genotyping (*15*), further supports nosocomial spread. Nonetheless, whole-genome sequencing is needed to confirm clonal transmission.

Hospital A, where most cases occurred, exhibited multiple epidemic peaks, suggesting persistent or recurrent environmental contamination or inadequate infection prevention and control measures. The development of concurrent clusters in hospitals from different cities also points to systemic weaknesses in infection control practices and limited local laboratory capacity for early fungal detection. Those findings emphasize the urgent need for strengthened hospital hygiene programs, environmental monitoring, and laboratory-based fungal surveillance at the national level.

Antifungal susceptibility testing revealed that nearly all isolates remained susceptible (wild-type) to amphotericin B, echinocandins, and azoles, consistent with previous reports describing *W. anomalus* as generally susceptible to conventional antifungal agents. However, when interpreted with the ECVs established for *C. albicans*, *W. anomalus* has higher MICs for flucytosine, itraconazole, voriconazole, and fluconazole (*1*,*20*,*22*). Therefore, the detection of isolates with elevated MICs to fluconazole and amphotericin B warrants attention. For agents like isavuconazole and anidulafungin, for which formal ECVs are not yet defined, we applied European Committee on Antimicrobial Susceptibility Testing cutoffs (0.25 µg/mL and 0.125 µg/mL), classifying all isolates in this analysis as wild-type (*23*). This overall susceptibility pattern contrasts with recent reports from other regions. A recent study from India reported that 50/70 (72%) *W. anomalus* isolates were non–wild-type for fluconazole, 17% demonstrated cross-resistance with voriconazole, and 1.4% demonstrated cross-resistance with micafungin (*24*). Furthermore, a pan–azole-resistant and pan–echinocandin-resistant *W. anomalus* bloodstream isolate has been recently described in China (*25*). Those findings suggest that while susceptibility remains common, the emergence of resistance, particularly multidrug resistance, poses a potential threat to clinical management, especially in resource-limited settings (*26*).

The MALDI-TOF mass spectrometry dendrogram corroborated those findings and revealed a strong association between clustering patterns and geographic origin, while also indicating potential epidemiologic links beyond individual healthcare institutions. Isolates from Los Teques and Valencia formed compact, highly similar clusters, whereas Caracas isolates were distributed across multiple branches. The detection of genetically and proteomically related isolates across different locations suggests possible shared sources, delayed recognition of transmission events, or gaps in local surveillance capacity. In addition to clustering, MALDI-TOF mass spectrometry successfully identified all isolates at the species level, confirming its reliability for fast and accurate identification of *W. anomalus*. Although MALDI-TOF mass spectrometry enabled rapid and reliable identification in this study, its availability and cost may limit use in resource-constrained settings such as Venezuela. In many hospitals, access remains restricted to reference laboratories, highlighting the need for more accessible and scalable diagnostic alternatives to support timely detection of fungal bloodstream infections.

The main limitation of this investigation was the limited access to clinical data from most cases. Strengthening data integration between clinical and laboratory systems would substantially improve the early detection and management of future fungal outbreaks. This outbreak highlights the capacity of uncommon yeasts, such as *W. anomalus*, to cause large-scale nosocomial infections under conditions of limited infection control and microbiological oversight. Continuous training of healthcare personnel, the adoption of rapid yeast identification tools (e.g., MALDI-TOF mass spectrometry), and the implementation of structured mycological surveillance networks are essential measures to mitigate similar events in the future.

In conclusion, this multicenter outbreak underscores the need for sustained vigilance for opportunistic yeasts in hospital settings, especially in neonatal and pediatric units. Strengthening diagnostic capacity, reinforcing infection prevention and control programs, and integrating fungal BSIs into national surveillance systems are critical to prevent recurrence and improve patient outcomes in Venezuela and similar settings.

Appendix Additional information about outbreak of *Wickerhamomyces anomalus* (formerly *Candida pelliculosa*) bloodstream infections, Venezuela, 2022–2023
