# Supplementary material for: Outbreak of Wickerhamomyces anomalus (formerly Candida pelliculosa) Bloodstream Infections, Venezuela, 2022–2023
Source: Emerg Infect Dis. 2026 Jun;32(6):933–40. doi: 10.3201/eid3206.251978 (PMC13245250; doi:10.3201/eid3206.251978)
Supplement: Appendix — Additional information about outbreak of Wickerhamomyces anomalus (formerly Candida pelliculosa) bloodstream infections, Venezuela, 2022–2023 [file 25-1978-Techapp-s1.pdf]

Article DOI: https://doi.org/10.3201/eid3206.251978

*EID cannot ensure accessibility for supplementary materials supplied by authors. Readers who have difficulty accessing supplementary content should contact the authors for assistance.*

# Outbreak of *Wickerhamomyces anomalus* (formerly *Candida pelliculosa*) Bloodstream Infections, Venezuela, 2022–2023

## Appendix

**Appendix Table 1.** Overview of 99 *Wickerhamomyces anomalus* bloodstream isolates, including source, date of collection and hospital.

| ID | Hospital | Age | Sex | Isolation date |
|---|---|---|---|---|
| **L-03** | A | 5 d | Male | 17/11/2022 |
| **L-04** | A | 15 d | Female | 17/11/2022 |
| **L-05** | A | 6 d | Male | 2/12/2022 |
| **L-06** | A | 1 mo | Male | 5/12/2022 |
| **L-07** | A | 1 mo | Male | 13/12/2022 |
| **L-08** | A | 10 d | Female | 14/12/2022 |
| **L-10** | A | 12 d | Female | 23/12/2022 |
| **L-12** | A | 9 d | Male | 13/1/2023 |
| **L-13** | A | 37 y | Male | 16/1/2023 |
| **L-15** | A | 7 d | Male | 27/1/2023 |
| **L-17** | A | 19 d | Male | 14/3/2023 |
| **L-19** | A | 23 d | Male | 22/3/2023 |
| **L-20** | A | 8 d | Male | 24/3/2023 |
| **L-21** | A | 9 d | Male | 7/2/2023 |
| **L-22** | A | 12 d | Male | 7/2/2023 |
| **L-26** | A | 9 d | Female | 16/2/2023 |
| **L-28** | A | 10 d | Female | 27/2/2023 |
| **L-30** | A | 6 d | Male | 28/2/2023 |
| **L-32** | A | 16 d | Male | 6/3/2023 |
| **L-33** | A | 9 d | Male | 31/3/2023 |
| **L-34** | A | 8 d | Male | 31/3/2023 |
| **L-37** | A | 6 d | Female | 10/4/2023 |
| **L-39** | F | 10 d | Female | 26/4/2023 |
| **L-40** | F | 10 d | Male | 26/4/2023 |
| **L-41** | A | 6 d | Female | 9/5/2023 |
| **L-42** | A | 8 d | Female | 9/5/2023 |
| **L-44** | A | 5 d | Female | 12/5/2023 |
| **L-45** | A | 6 mo | Male | 13/5/2023 |
| **L-48** | A | 8 d | Male | 22/5/2023 |
| **L-49** | A | 8 d | Male | 22/5/2023 |
| **L-50** | A | 21 d | Female | 22/5/2023 |
| **L-54** | A | 10 d | Male | 5/6/2023 |
| **L-55** | A | 5 d | Male | 6/6/2023 |
| **L-56** | A | 22 d | Male | 6/6/2023 |
| **L-59** | A | 8 d | Male | 6/6/2023 |
| **L-62** | A | 13 d | Male | 13/6/2023 |
| **L-64** | B | 3 y | Male | 7/6/2023 |
| **L-69** | A | 3 d | Male | 23/6/2023 |
| **L-106** | A | 17 d | Male | 20/9/2022 |
| **L-212** | A | 24 d | Female | 6/7/2023 |
| **L-214** | A | 9 d | Female | 11/7/2023 |
| **L-236** | A | 5 d | Male | 25/7/2023 |

| ID | Hospital | Age | Sex | Isolation date |
|---|---|---|---|---|
| **L-274** | A | 14 d | Female | 3/8/2023 |
| **L-277** | A | 7 d | Male | 31/7/2023 |
| **L-278** | A | 10 d | Male | 3/8/2023 |
| **L-279** | A | 11 d | Male | 1/8/2023 |
| **L-280** | A | 13 d | Female | 3/8/2023 |
| **L-281** | A | 11 d | Female | 1/8/2023 |
| **L-282** | A | 15 d | Female | 3/8/2023 |
| **L-283** | A | 10 d | Male | 3/8/2023 |
| **L-292** | A | 26 d | Female | 14/8/2023 |
| **L-311** | A | 7 d | Female | 25/8/2023 |
| **L-322** | A | 8 d | Male | 17/8/2023 |
| **L-323** | A | 7 d | Male | 17/8/2023 |
| **L-325** | D | 7 d | Male | 11/8/2023 |
| **L-326** | D | 3 y | Male | 11/8/2023 |
| **L-332** | G | 26 d | Female | 28/4/2023 |
| **L-333** | G | 5 d | Male | 28/4/2023 |
| **L-334** | H | 2 mo | Female | 28/4/2023 |
| **L-335** | G | 4 d | Female | 28/4/2023 |
| **L-336** | G | 10 mo | Female | 28/4/2023 |
| **L-337** | G | 3 y | Female | 28/4/2023 |
| **L-339** | D | 3 y | Male | 18/8/2023 |
| **L-341** | A | 19 d | Female | 6/9/2023 |
| **L-343** | G | 4 d | Female | 28/4/2023 |
| **L-344** | G | 2 d | Male | 28/4/2023 |
| **L-348** | G | 9 d | Male | 28/4/2023 |
| **L-347** | G | 4 y | Male | 28/4/2023 |
| **L-350** | G | 18 d | Male | 28/4/2023 |
| **L-358** | A | 8 d | Female | 8/9/2023 |
| **L-360** | F | 7 d | Female | 11/9/2023 |
| **L-361** | A | 9 d | Female | 13/9/2023 |
| **L-363** | E | 1 mo | Male | 14/9/2023 |
| **L-396** | A | 21 d | Female | 17/10/2023 |
| **L-397** | F | 12 d | Male | 10/10/2023 |
| **L-398** | F | 13 d | Male | 10/10/2023 |
| **L-399** | A | 2 mo | Male | 11/10/2023 |
| **L-400** | E | 30 d | Male | 9/10/2023 |
| **L-401** | A | 30 d | Male | 13/10/2023 |
| **L-402** | E | 1 mo | Male | 21/9/2023 |
| **L-404** | A | 10 d | Female | 8/9/2023 |
| **L-405** | A | 15 d | Male | 28/9/2023 |
| **L-406** | A | 17 d | Male | 25/9/2023 |
| **L-408** | F | 15 d | Female | 4/10/2023 |
| **L-410** | A | 23 d | Male | 2/10/2023 |
| **L-411** | A | 9 d | Male | 4/10/2023 |
| **L-412** | A | 8 d | Male | 4/10/2023 |
| **L-414** | F | 7 d | Female | 20/9/2023 |
| **L-415** | A | 17 d | Male | 20/9/2023 |
| **L-416** | C | 23 y | Female | 25/9/2023 |
| **L-417** | A | 22 d | Female | 21/9/2023 |
| **L-418** | A | 1 mo | Male | 23/10/2023 |
| **L-419** | A | 2 d | Male | 24/10/2023 |
| **L-420** | A | 26 d | Female | 23/10/2023 |
| **L-421** | A | 1 mo | Male | 23/10/2023 |
| **L-423** | A | 1 mo | Male | 26/10/2023 |
| **L-426** | A | 2 mo | Male | 31/10/2023 |
| **L-427** | F | 15 d | Female | 31/10/2023 |
| **L-428** | F | 11 d | Female | 31/10/2023 |

**Appendix Table 2.** In vitro MICs (MICs) of 99 *Wickerhamomyces anomalus* isolates against eight antifungals by using CLSI M27 broth microdilution. MICs in µg/mL.

| ID | AMB | FLU | ITC | VOR | POS | ISA | AFG | MFG |
|---|---|---|---|---|---|---|---|---|
| **L-03** | 0.063 | 1 | 0.125 | 0.25 | 0.125 | 0.125 | ≤0.008 | ≤0.008 |
| **L-04** | 0.063 | 2 | 0.125 | 0.125 | 0.125 | 0.063 | ≤0.008 | ≤0.008 |
| **L-05** | 0.063 | 2 | 0.063 | 0.063 | 0.063 | 0.031 | ≤0.008 | 0.016 |
| **L-06** | 0.063 | 2 | 0.125 | 0.125 | 0.063 | 0.031 | ≤0.008 | 0.031 |
| **L-07** | 0.063 | 2 | 0.063 | 0.063 | 0.063 | 0.031 | 0.016 | ≤0.008 |
| **L-08** | 0.063 | 2 | 0.063 | 0.063 | 0.063 | 0.031 | ≤0.008 | ≤0.008 |
| **L-10** | 0.063 | 2 | 0.063 | 0.063 | 0.063 | 0.031 | ≤0.008 | 0.016 |
| **L-12** | 0.063 | 2 | 0.063 | 0.063 | 0.063 | 0.031 | ≤0.008 | ≤0.008 |
| **L-13** | 0.063 | 8 | 0.25 | 0.125 | 0.25 | 0.125 | ≤0.008 | ≤0.008 |
| **L-15** | 0.063 | 2 | 0.063 | 0.063 | 0.031 | 0.031 | ≤0.008 | ≤0.008 |
| **L-17** | 0.063 | 2 | 0.063 | 0.031 | 0.031 | 0.031 | 0.016 | 0.016 |
| **L-19** | 0.063 | 2 | 0.063 | 0.063 | 0.063 | 0.031 | 0.016 | 0.031 |
| **L-20** | 0.063 | 2 | 0.063 | 0.063 | 0.125 | 0.031 | 0.016 | 0.031 |
| **L-21** | 0.063 | 2 | 0.063 | 0.063 | 0.063 | 0.063 | 0.016 | 0.016 |
| **L-22** | 0.063 | 2 | 0.063 | 0.063 | 0.063 | 0.031 | ≤0.008 | ≤0.008 |
| **L-26** | 0.063 | 2 | 0.063 | 0.063 | 0.063 | 0.031 | 0.016 | 0.016 |
| **L-28** | 0.063 | 2 | 0.063 | 0.063 | 0.063 | 0.031 | ≤0.008 | ≤0.008 |
| **L-30** | 0.063 | 2 | 0.063 | 0.063 | 0.063 | ≤0.016 | ≤0.008 | ≤0.008 |
| **L-32** | 0.063 | 2 | 0.063 | 0.063 | 0.063 | 0.031 | ≤0.008 | ≤0.008 |
| **L-33** | 0.063 | 2 | 0.063 | 0.063 | 0.031 | ≤0.016 | 0.016 | 0.016 |
| **L-34** | 0.125 | 2 | 0.063 | 0.063 | 0.063 | 0.031 | 0.016 | ≤0.008 |
| **L-37** | 0.063 | 2 | 0.063 | 0.063 | 0.063 | ≤0.016 | ≤0.008 | 0.016 |
| **L-39** | 0.063 | 1 | 0.125 | 0.063 | 0.063 | 0.063 | ≤0.008 | ≤0.008 |
| **L-40** | 0.063 | 2 | 0.125 | 0.063 | 0.063 | 0.031 | 0.016 | 0.016 |
| **L-41** | 0.063 | 2 | 0.063 | 0.063 | 0.063 | ≤0.016 | ≤0.008 | ≤0.008 |
| **L-42** | 0.125 | 2 | 0.063 | 0.063 | 0.031 | ≤0.016 | ≤0.008 | ≤0.008 |
| **L-44** | 0.063 | 2 | 0.063 | 0.063 | 0.031 | 0.031 | ≤0.008 | ≤0.008 |
| **L-45** | 0.063 | 1 | 0.031 | 0.031 | 0.063 | ≤0.016 | ≤0.008 | ≤0.008 |
| **L-48** | 0.063 | 2 | 0.063 | 0.125 | 0.031 | 0.031 | 0.016 | ≤0.008 |
| **L-49** | 0.063 | 2 | 0.063 | 0.063 | 0.031 | 0.031 | ≤0.008 | ≤0.008 |
| **L-50** | 0.063 | 4 | 0.063 | 0.063 | 0.063 | 0.031 | ≤0.008 | ≤0.008 |
| **L-54** | 0.125 | 2 | 0.125 | 0.063 | 0.063 | ≤0.016 | 0.016 | ≤0.008 |
| **L-55** | 0.063 | 2 | 0.063 | 0.063 | 0.063 | ≤0.016 | ≤0.008 | 0.016 |
| **L-56** | 0.063 | 2 | 0.063 | 0.063 | 0.063 | 0.031 | ≤0.008 | 0.016 |
| **L-59** | 0.063 | 2 | 0.063 | 0.063 | 0.063 | 0.031 | ≤0.008 | ≤0.008 |
| **L-62** | 0.063 | 2 | 0.063 | 0.063 | 0.063 | 0.031 | ≤0.008 | ≤0.008 |
| **L-64** | 0.063 | 2 | 0.063 | 0.063 | 0.031 | 0.031 | ≤0.008 | ≤0.008 |
| **L-69** | 0.063 | 4 | 0.125 | 0.125 | 0.125 | 0.125 | ≤0.008 | 0.031 |
| **L-106** | 0.063 | 1 | 0.063 | 0.125 | 0.063 | 0.063 | ≤0.008 | ≤0.008 |
| **L-212** | 0.063 | 2 | 0.063 | 0.063 | 0.063 | 0.031 | ≤0.008 | ≤0.008 |
| **L-214** | 0.063 | 2 | 0.063 | 0.063 | 0.031 | 0.031 | 0.016 | 0.016 |
| **L-236** | 0.063 | 2 | 0.063 | 0.063 | 0.063 | 0.031 | 0.031 | 0.031 |
| **L-274** | 0.063 | 2 | 0.063 | 0.063 | 0.063 | 0.031 | 0.016 | 0.016 |
| **L-277** | 0.063 | 2 | 0.063 | 0.063 | 0.063 | ≤0.016 | ≤0.008 | ≤0.008 |
| **L-278** | 0.063 | 2 | 0.063 | 0.063 | 0.063 | 0.031 | ≤0.008 | ≤0.008 |
| **L-279** | 0.063 | 2 | 0.063 | 0.063 | 0.063 | 0.031 | ≤0.008 | 0.016 |
| **L-280** | 0.063 | 2 | 0.063 | 0.063 | 0.063 | 0.031 | ≤0.008 | ≤0.008 |
| **L-281** | 0.125 | 2 | 0.063 | 0.031 | 0.063 | ≤0.016 | 0.016 | 0.031 |
| **L-282** | 0.125 | 2 | 0.063 | 0.031 | 0.031 | ≤0.016 | ≤0.008 | ≤0.008 |
| **L-283** | 0.063 | 4 | 0.063 | 0.063 | 0.063 | 0.031 | ≤0.008 | ≤0.008 |
| **L-292** | 0.063 | 2 | 0.125 | 0.125 | 0.063 | 0.031 | ≤0.008 | ≤0.008 |
| **L-311** | 0.063 | 2 | 0.063 | 0.063 | 0.063 | 0.031 | ≤0.008 | ≤0.008 |
| **L-322** | 0.063 | 1 | 0.063 | 0.125 | 0.125 | 0.063 | ≤0.008 | ≤0.008 |
| **L-323** | 0.063 | 2 | 0.063 | 0.063 | 0.063 | 0.031 | 0.016 | ≤0.008 |
| **L-325** | 0.125 | 4 | 0.063 | 0.125 | 0.063 | 0.063 | ≤0.008 | 0.016 |
| **L-326** | 0.063 | 2 | 0.063 | 0.031 | 0.031 | ≤0.016 | ≤0.008 | ≤0.008 |
| **L-332** | 0.063 | 2 | 0.063 | 0.063 | 0.063 | 0.031 | ≤0.008 | ≤0.008 |
| **L-333** | 0.125 | 1 | 0.063 | 0.063 | 0.063 | 0.031 | ≤0.008 | ≤0.008 |
| **L-334** | 0.063 | 1 | 0.063 | 0.063 | 0.063 | 0.031 | ≤0.008 | 0.063 |
| **L-335** | 0.25 | 2 | 0.063 | 0.063 | 0.063 | 0.031 | 0.016 | 0.031 |
| **L-336** | 0.063 | 2 | 0.031 | 0.031 | 0.031 | ≤0.016 | ≤0.008 | ≤0.008 |
| **L-337** | 0.125 | 4 | 0.063 | 0.063 | 0.063 | 0.031 | ≤0.008 | 0.031 |
| **L-339** | 0.25 | 2 | 0.031 | 0.031 | 0.063 | 0.031 | 0.031 | 0.063 |
| **L-341** | 0.063 | 2 | 0.063 | 0.063 | 0.063 | ≤0.016 | ≤0.008 | ≤0.008 |
| **L-343** | 0.125 | 2 | 0.063 | 0.063 | 0.031 | 0.031 | ≤0.008 | 0.016 |
| **L-344** | 0.063 | 2 | 0.125 | 0.063 | 0.063 | ≤0.016 | ≤0.008 | 0.016 |
| **L-348** | 0.125 | 2 | 0.063 | 0.063 | 0.063 | 0.031 | 0.031 | 0.016 |

| ID | AMB | FLU | ITC | VOR | POS | ISA | AFG | MFG |
|---|---|---|---|---|---|---|---|---|
| **L-347** | 0.063 | 2 | 0.125 | 0.125 | 0.063 | 0.031 | 0.016 | ≤0.008 |
| **L-350** | 0.125 | 2 | 0.063 | 0.063 | 0.031 | 0.031 | ≤0.008 | 0.016 |
| **L-358** | 0.063 | 2 | 0.063 | 0.063 | 0.063 | 0.031 | 0.016 | 0.016 |
| **L-360** | 0.125 | 2 | 0.063 | 0.063 | 0.063 | 0.031 | 0.031 | 0.016 |
| **L-361** | 0.063 | 2 | 0.063 | 0.063 | 0.063 | 0.031 | ≤0.008 | ≤0.008 |
| **L-363** | 0.063 | 1 | 0.063 | 0.031 | 0.063 | ≤0.016 | ≤0.008 | 0.016 |
| **L-396** | 0.063 | 2 | 0.063 | 0.063 | 0.063 | ≤0.016 | ≤0.008 | ≤0.008 |
| **L-397** | 0.25 | 2 | 0.063 | 0.063 | 0.063 | 0.063 | ≤0.008 | ≤0.008 |
| **L-398** | 0.25 | 2 | 0.063 | 0.063 | 0.063 | 0.031 | ≤0.008 | ≤0.008 |
| **L-399** | 0.25 | 2 | 0.063 | 0.063 | 0.063 | ≤0.016 | 0.016 | 0.016 |
| **L-400** | 0.125 | 2 | 0.063 | 0.063 | 0.063 | 0.031 | 0.031 | 0.031 |
| **L-401** | 0.125 | 1 | 0.063 | 0.063 | 0.031 | ≤0.016 | 0.016 | 0.016 |
| **L-402** | 0.063 | 2 | 0.063 | 0.063 | 0.031 | 0.031 | 0.016 | ≤0.008 |
| **L-404** | 0.063 | 1 | 0.063 | 0.031 | 0.063 | ≤0.016 | ≤0.008 | ≤0.008 |
| **L-405** | 0.125 | 2 | 0.063 | 0.063 | 0.031 | ≤0.016 | 0.031 | 0.031 |
| **L-406** | 0.063 | 2 | 0.125 | 0.125 | 0.125 | 0.031 | 0.016 | 0.016 |
| **L-408** | 0.125 | 2 | 0.063 | 0.063 | 0.063 | 0.031 | ≤0.008 | ≤0.008 |
| **L-410** | 0.063 | 2 | 0.063 | 0.063 | 0.063 | 0.031 | 0.016 | 0.016 |
| **L-411** | 0.063 | 4 | 0.125 | 0.125 | 0.063 | 0.063 | ≤0.008 | ≤0.008 |
| **L-412** | 0.063 | 4 | 0.063 | 0.063 | 0.031 | ≤0.016 | 0.016 | ≤0.008 |
| **L-414** | 0.063 | 2 | 0.063 | 0.031 | 0.063 | 0.031 | ≤0.008 | ≤0.008 |
| **L-415** | 0.125 | 4 | 0.125 | 0.063 | 0.125 | 0.063 | ≤0.008 | ≤0.008 |
| **L-416** | 0.063 | 2 | 0.063 | 0.063 | 0.063 | ≤0.016 | ≤0.008 | 0.016 |
| **L-417** | 0.063 | 2 | 0.063 | 0.063 | 0.063 | 0.031 | 0.016 | 0.031 |
| **L-418** | 0.063 | 2 | 0.063 | 0.063 | 0.063 | 0.031 | ≤0.008 | 0.016 |
| **L-419** | 0.063 | 2 | 0.063 | 0.063 | 0.063 | ≤0.016 | ≤0.008 | ≤0.008 |
| **L-420** | 0.063 | 2 | 0.063 | 0.063 | 0.063 | 0.031 | ≤0.008 | ≤0.008 |
| **L-421** | 0.063 | 2 | 0.063 | 0.063 | 0.063 | 0.031 | ≤0.008 | ≤0.008 |
| **L-423** | 0.125 | 1 | 0.031 | 0.031 | 0.031 | ≤0.016 | ≤0.008 | ≤0.008 |
| **L-426** | 0.063 | 2 | 0.063 | 0.063 | 0.125 | 0.031 | ≤0.008 | ≤0.008 |
| **L-427** | 0.125 | 2 | 0.063 | 0.063 | 0.031 | ≤0.016 | ≤0.008 | ≤0.008 |
| **L-428** | 0.063 | 2 | 0.125 | 0.063 | 0.063 | ≤0.016 | 0.031 | 0.031 |

**AMB: amphotericin B; FLU: fluconazole; ITC: itraconazole; VOR: voriconazole; POS: posaconazole; ISA: isavuconazole; AFG: anidulafungin; MFG: micafungin.**
